# Correction: Estimation of enteric methane emissions in dairy cows under grazing a silvopastoral system and a grass monoculture in the Colombian Amazonian foothills

**DOI:** 10.1371/journal.pone.0344686

**Published:** 2026-03-10

**Authors:** Juan Pablo Narváez-Herrera, Joaquín Angulo-Arizala, Wilson Andrés Barragán-Hernández, Liliana Mahecha-Ledesma

The following information is missing from the Funding statement: The authors also acknowledge Universidad de Antioquia for supporting the open access publication of this article through its transformative publishing agreement with PLOS (Consorcio Colombia–PLOS).
