# Supplementary material for: Estimation of enteric methane emissions in dairy cows under grazing a silvopastoral system and a grass monoculture in the Colombian Amazonian foothills
Source: PLoS One. 2026 Jan 30;21(1):e0337719. doi: 10.1371/journal.pone.0337719 (PMC12857995; doi:10.1371/journal.pone.0337719)
Supplement: S1 Table — (DOCX) [file pone.0337719.s001.docx]

**PLOS One**

**Estimation of enteric methane emissions in dairy cows under grazing a silvopastoral system and a grass monoculture in the Colombian Amazonian foothills**

**Supplementary Table S1.** Characteristics of Experimental Animals

| **Group** | **Weight (kg)** | **Number of Calvings** | **Days in Milk (DIM)** | **Milk Production (L/cow/day)** | **Milk Fat (%)** | **Milk Protein (%)** | **Body Condition Score (BCS)** |
| --- | --- | --- | --- | --- | --- | --- | --- |
| 1 | 443 ± 21.35 | 3.2 ± 2.64 | 120 ± 32.86 | 14.2 ± 3.6 | 4.1 ± 0.07 | 3.34 ± 0.12 | 3.00 ± 0.25 |
| 2 | 462 ± 16.00 | 3.2 ± 1.60 | 126 ± 29.39 | 13.8 ± 1.6 | 4.0 ± 0.14 | 3.34 ± 0.14 | 3.25 ± 0.29 |

Values represent the means and standard deviations of the last ten days prior to the start of the experiment.
