# Supplementary material for: Estimation of enteric methane emissions in dairy cows under grazing a silvopastoral system and a grass monoculture in the Colombian Amazonian foothills
Source: PLoS One. 2026 Jan 30;21(1):e0337719. doi: 10.1371/journal.pone.0337719 (PMC12857995; doi:10.1371/journal.pone.0337719)
Supplement: Attachment — Submitted filename: PONE-D-25-33619.pdf [file pone.0337719.s004.pdf]

PLOS ONE

Estimation of Enteric Methane Emissions in Dual-purpose Cows under grazing a Silvopastoral system and a Grass Monoculture in the Colombian Amazonian Foothills

--Manuscript Draft--

**Manuscript Number:**	PONE-D-25-33619
**Article Type:**	Research Article
**Full Title:**	Estimation of Enteric Methane Emissions in Dual-purpose Cows under grazing a Silvopastoral system and a Grass Monoculture in the Colombian Amazonian Foothills
**Short Title:**	Methane Emissions in Dual-purpose Cows under Grazing
**Corresponding Author:**	Liliana Mahecha-Ledesma, Ph.D<br>University of Antioquia: Universidad de Antioquia<br>COLOMBIA
**Keywords:**	Keywords: Greenhouse gas emissions; grazing systems; livestock smart; Methane intensity; sustainability
**Abstract:**	Mitigating enteric methane emissions without compromising productivity remains a major challenge in tropical livestock production systems. This study assessed the potential of a laser methane detector (LMD) to estimate emissions in dual-purpose cows managed under two grazing systems: a silvopastoral system (SPS) with tree and shrub species, and a traditional pasture (TP) based on grass monoculture. A crossover design was applied with two groups of five animals, and emissions were measured over four periods. Daily methane emissions were significantly lower in the SPS (233 ± 6.95 g CH_4/animal/day) compared to the TP (277 ± 8.87 g; $p < 0.0001$). Similarly, methane intensity per unit of output was reduced in the SPS across all metrics: per kg of milk (15.5 vs. 20.7), ECM (16.0 vs. 21.2), and DMI (18.9 vs. 26.7 g CH_4; $p < 0.0001$). Animal activity had a significant effect, with higher emissions recorded during grazing and ruminating, and lower values during resting and milking ($p < 0.05$). The use of the LMD allowed differentiation among activities, confirming its utility as a non-invasive tool for field studies. These findings support the adoption of silvopastoral systems as a viable strategy for reducing greenhouse gas emissions in tropical cattle production without compromising animal performance.
**Order of Authors:**	JUAN PABLO NARVAEZ HERRERA, Ph.D
	Joaquín Angulo-Arizala, Ph.D
	Wilson Andrés Barragán-Hernández, Ph.D
	Liliana Mahecha-Ledesma, Ph.D
**Additional Information:**	
**Question**	**Response**
**Financial Disclosure**<br><br>Enter a financial disclosure statement that describes the sources of funding for the work included in this submission. Review the submission guidelines for detailed requirements. View published research articles from *PLOS ONE* for specific examples.<br><br>This statement is required for submission and **will appear in the published article** if the submission is accepted. Please make	Yes

Powered by Editorial Manager® and ProduXion Manager® from Aries Systems Corporation

sure it is accurate.<br><br>**Funded studies**<br>Enter a statement with the following details:<br>• Initials of the authors who received each award<br>• Grant numbers awarded to each author<br>• The full name of each funder<br>• URL of each funder website<br>• Did the sponsors or funders play any role in the study design, data collection and analysis, decision to publish, or preparation of the manuscript?<br><br>Did you receive funding for this work?	
Please add funding details.<br>as follow-up to "**Financial Disclosure**<br><br>Enter a financial disclosure statement that describes the sources of funding for the work included in this submission. Review the submission guidelines for detailed requirements. View published research articles from *PLOS ONE* for specific examples.<br><br>This statement is required for submission and **will appear in the published article** if the submission is accepted. Please make sure it is accurate.<br><br>**Funded studies**<br>Enter a statement with the following details:<br>• Initials of the authors who received each award<br>• Grant numbers awarded to each author<br>• The full name of each funder<br>• URL of each funder website<br>• Did the sponsors or funders play any role in the study design, data collection and analysis, decision to publish, or preparation of the manuscript?<br><br>Did you receive funding for this work?"	JPNH received funding from the Ministry of Science, Technology, and Innovation of Colombia through the Bicentennial Excellence Doctoral Scholarship Program - Cohort II (Doctoral Scholarship Agreement No. 20230030-20-21).
Please select the country of your main research funder (please select carefully	COLOMBIA - CO

as in some cases this is used in fee calculation).<br>as follow-up to "**Financial Disclosure**<br><br>Enter a financial disclosure statement that describes the sources of funding for the work included in this submission. Review the submission guidelines for detailed requirements. View published research articles from *PLOS ONE* for specific examples.<br><br>This statement is required for submission and **will appear in the published article** if the submission is accepted. Please make sure it is accurate.<br><br>**Funded studies**<br>Enter a statement with the following details:<br>• Initials of the authors who received each award<br>• Grant numbers awarded to each author<br>• The full name of each funder<br>• URL of each funder website<br>• Did the sponsors or funders play any role in the study design, data collection and analysis, decision to publish, or preparation of the manuscript?<br><br>Did you receive funding for this work?"	
**Competing Interests**<br><br>Use the instructions below to enter a competing interest statement for this submission. On behalf of all authors, disclose any competing interests that could be perceived to bias this work—acknowledging all financial support and any other relevant financial or non-financial competing interests.<br><br>This statement is required for submission and **will appear in the published article** if the submission is accepted. Please make sure it is accurate and that any funding sources listed in your Funding Information later in the submission form are also	The authors have declared that no competing interests exist.

Powered by Editorial Manager® and ProduXion Manager® from Aries Systems Corporation

declared in your Financial Disclosure statement.<br><br>View published research articles from *PLOS ONE* for specific examples.<br><br>**NO authors have competing interests**<br><br>Enter: *The authors have declared that no competing interests exist.*<br><br>**Authors with competing interests**<br><br>Enter competing interest details beginning with this statement:<br><br>*I have read the journal's policy and the authors of this manuscript have the following competing interests: [insert competing interests here]*<br><br>* typeset	
**Ethics Statement**<br><br>Enter an ethics statement for this submission. This statement is required if the study involved:<br><br>• Human participants<br>• Human specimens or tissue<br>• Vertebrate animals or cephalopods<br>• Vertebrate embryos or tissues<br>• Field research<br><br>Write "N/A" if the submission does not require an ethics statement.<br><br>General guidance is provided below. Consult the submission guidelines for detailed instructions. **Make sure that all information entered here is included in the Methods section of the manuscript.**	All procedures involving animals were conducted in accordance with protocols approved by the Ethics Committee for Animal Experimentation (Comité de Ética para la Experimentación Animal, CEEA) of the University of Antioquia. The study was approved under Act No. 0146, dated June 7, 2022. All animal handling complied with institutional guidelines to ensure welfare and minimize distress. No animals were subjected to anesthesia, euthanasia, or invasive procedures during this study.

**Format for specific study types**<br><br>**Human Subject Research (involving human participants and/or tissue)**<br>• Give the name of the institutional review board or ethics committee that approved the study<br>• Include the approval number and/or a statement indicating approval of this research<br>• Indicate the form of consent obtained (written/oral) or the reason that consent was not obtained (e.g. the data were analyzed anonymously)<br><br>**Animal Research (involving vertebrate animals, embryos or tissues)**<br>• Provide the name of the Institutional Animal Care and Use Committee (IACUC) or other relevant ethics board that reviewed the study protocol, and indicate whether they approved this research or granted a formal waiver of ethical approval<br>• Include an approval number if one was obtained<br>• If the study involved *non-human primates*, add *additional details* about animal welfare and steps taken to ameliorate suffering<br>• If anesthesia, euthanasia, or any kind of animal sacrifice is part of the study, include briefly which substances and/or methods were applied<br><br>**Field Research**<br><br>Include the following details if this study involves the collection of plant, animal, or other materials from a natural setting:<br>• Field permit number<br>• Name of the institution or relevant body that granted permission	
**Data Availability**<br><br>Authors are required to make all data underlying the findings described fully available, without restriction, and from the time of publication. PLOS allows rare exceptions to address legal and ethical concerns. See the PLOS Data Policy and FAQ for detailed information.	Yes - all data are fully available without restriction

A Data Availability Statement describing where the data can be found is required at submission. Your answers to this question constitute the Data Availability Statement and **will be published in the article**, if accepted.<br><br>**Important:** Stating 'data available on request from the author' is not sufficient. If your data are only available upon request, select 'No' for the first question and explain your exceptional situation in the text box.<br><br>Do the authors confirm that all data underlying the findings described in their manuscript are fully available without restriction?	
**Describe where the data may be found in full sentences. If you are copying our sample text, replace any instances of XXX with the appropriate details.**<br><br>• If the data are **held or will be held in a public repository**, include URLs, accession numbers or DOIs. If this information will only be available after acceptance, indicate this by ticking the box below. For example: *All XXX files are available from the XXX database (accession number(s) XXX, XXX.).*<br>• If the data are all contained **within the manuscript and/or Supporting Information files**, enter the following: *All relevant data are within the manuscript and its Supporting Information files.*<br>• If neither of these applies but you are able to provide **details of access elsewhere**, with or without limitations, please do so. For example:<br><br>*Data cannot be shared publicly because of [XXX]. Data are available from the XXX Institutional Data Access / Ethics Committee (contact via XXX) for researchers who meet the criteria for access to confidential data.*<br><br>*The data underlying the results presented in the study are available from (include the name of the third party*	All relevant data are within the manuscript and its Supporting Information files.

Powered by Editorial Manager® and ProduXion Manager® from Aries Systems Corporation

and contact information or URL).<br>• This text is appropriate if the data are owned by a third party and authors do not have permission to share the data.<br><br>* typeset	
Additional data availability information:	

Manuscript
Click here to access/download;Manuscript;renamed_a4bbc.docx

Estimation of Enteric Methane Emissions in Dual-purpose Cows under grazing a Silvopastoral system and a Grass Monoculture in the Colombian Amazonian Foothills

Juan Pablo Narváez-Herrera[1]
Orcid: https://orcid.org/0000-0003-2766-2939
Email: juan.narvaezh@udea.edu.co

Co-authors

Joaquín Angulo-Arizala[1]
Orcid: https://orcid.org/0000-0003-3352-8795

Wilson Andrés Barragán-Hernández[2]
Orcid: https://orcid.org/0000-0003-3528-4296

Liliana Mahecha-Ledesma[1*]
Orcid: https://orcid.org/0000-0003-3377-8399
Email: liliana.mahecha@udea.edu.co

* Corresponding author

[1] Universidad de Antioquia, Facultad de Ciencias Agrarias, Grupo de Investigación en Ciencias Agrarias (GRICA). AA1126, Ciudadela Robledo, Calle 70 No 52-21. Medellín, Colombia
[2] Corporación Colombiana de Investigación Agropecuaria (AGROSAVIA), Centro de Investigación El Nus. San Roque, Colombia

Acknowledgments
The authors express their gratitude to the Universidad de Antioquia, the National Learning Service (SENA), the Agroforestry and Aquaculture Center Arapaima of the Putumayo Regional, the owners of La Primavera Farm, Mr. Aquilino Giraldo and Ms. Luz Marina Mejia, their family, and the Agricultural Sciences Research Group (GRICA), Sustainable Animal Production Systems Line.

Author Contributions: Study conception and design: J.P.N.-H., J.A.-A., W.B.-H., and L.M.-L.; field data collection: J.P.N.-H., Y.M.R.-G.; writing—original draft: J.P.N.-H.; manuscript critical revision: J.A.-A., W.B.-H., and L.M.-L. All authors reviewed and approved the final version of the manuscript.

Abstract

Mitigating enteric methane emissions without compromising productivity remains a major challenge in tropical livestock production systems. This study assessed the potential of a laser methane detector (LMD) to estimate emissions in dual-purpose cows managed under two grazing systems: a silvopastoral system (SPS) with tree and shrub species, and a traditional pasture (TP) based on grass monoculture. A crossover design was applied with two groups of five animals, and emissions were measured over four periods. Daily methane emissions were significantly lower in the SPS (233 ± 6.95 g CH_4/animal/day) compared to the TP (277 ± 8.87 g; $p < 0.0001$). Similarly, methane intensity per unit of output was reduced in the SPS across all metrics: per kg of milk (15.5 vs. 20.7), ECM (16.0 vs. 21.2), and DMI (18.9 vs. 26.7 g CH_4; $p < 0.0001$). Animal activity had a significant effect, with higher emissions recorded during grazing and ruminating, and lower values during resting and milking ($p < 0.05$). The use of the LMD allowed differentiation among activities, confirming its utility as a non-invasive tool for field studies. These findings support the adoption of silvopastoral systems as a viable strategy for reducing greenhouse gas emissions in tropical cattle production without compromising animal performance.

Keywords: Greenhouse gas emissions; grazing systems; livestock smart; Methane intensity; sustainability

1. Introduction

Livestock production is a key sector for global food security, but it is also one of the main sources of greenhouse gas (GHG) emissions, particularly methane (CH_4) generated through enteric fermentation in ruminants. It is estimated that livestock accounts for approximately 44% of global agricultural CH_4 emissions (Eismann et al., 2024). Methane has a global warming potential 28 times greater than that of carbon dioxide (CO_2) over a 100-year time horizon (Hammond et al., 2016), which underscores the urgent need for accessible quantification methods and effective mitigation strategies.

Methanogenesis in the rumen is a microbial process that converts hydrogen and CO_2 into CH_4, is primarily released into the atmosphere through belching (Hai et al., 2025). Several factors influence CH_4 production, including diet, ruminal microbiota composition, and host genetics (Azevedo et al., 2024). In this context, accurate quantification of enteric emissions is essential for assessing the environmental impact of livestock systems and for designing effective mitigation strategies.

Traditionally, CH_4 emissions have been measured using respiration chambers, the sulfur hexafluoride (SF_6) tracer technique, and portable systems such as GreenFeed (Hammond et al., 2015). While the SF_6 technique is applicable under grazing conditions and allows for individual animal monitoring, it requires the insertion of sampling equipment and tracer release devices, making it technically demanding and logistically complex to implement (Grainger et al., 2007). Moreover, SF_6 is a potent greenhouse gas with a global warming potential 23,500 times higher than CO_2, raising environmental concerns regarding its routine use (IPCC, 2021). Respiration chambers, though highly accurate, are invasive and restrict animal movement. GreenFeed systems, on the other hand, require animal habituation and concentrate feeding, which may not reflect typical grazing behavior (Ma et al., 2024).

In recent years, the laser methane detector (LMD) has emerged as a viable alternative for non-invasive, real-time measurement of CH_4 emissions under field conditions (Pereira et al., 2023). Studies have shown that the LMD can provide accurate estimates of emissions from grazing animals without interfering with their natural behavior (Meo Zilio et al., 2024). Nonetheless, measurement variability has been reported and may be influenced by factors such as sensor positioning, distance from the animal, and environmental conditions (Nunes et al., 2024).

In tropical regions such as the Colombian Amazonian foothills, livestock production is predominantly based on extensive grazing, with increasing interest in silvopastoral systems (SPS). These systems have demonstrated potential for CH_4 mitigation by improving diet quality and nutrient conversion efficiency in ruminants (Monteiro et al., 2024). The inclusion of tree and shrub species containing secondary metabolites may modulate ruminal fermentation and reduce CH_4 production (Delagarde et al., 2024). The present study aimed to evaluate the use of the laser methane detector (LMD) for estimating enteric CH_4 emissions in dual-purpose cows under grazing a silvopastoral system and a grass monoculture in the Colombian Amazonian foothills. The findings

are expected to contribute to the development of practical mitigation strategies for tropical livestock systems and to enhance the accuracy of GHG inventories in the region.

2. Materials and Methods

2.1 Study site

The study was conducted in the village of Aguanegra, located in the rural area of Puerto Asís, in the department of Putumayo, Colombia (0°33′09″N, 76°30′55″W), at an altitude of 270 meters above sea level. The region has a humid tropical climate, with an average annual temperature of 29 °C, relative humidity of 86%, and mean annual precipitation of 3,355 mm. Soils in the study area are classified as Ultisols with an average pH of 4.89 (IGAC, 2024). According to Holdridge's life zone classification (1966), the study site corresponds to tropical humid forest.

A total of ten lactating crossbred cows (Bos taurus × Bos indicus), of undefined Holstein and Gyr lineage, were selected. Animals were chosen based on minimal variation in average milk yield over the preceding ten days (MY), milk protein and fat content, days in milk (DIM), parity number (PN), and body weight (BW). Based on these criteria, two homogeneous groups of animals were formed for the evaluation (Table 1).

The experimental period lasted 76 days, divided into four 19-day periods, each consisting of 14 days of adaptation to the respective grazing system silvopastoral system (SPS) or traditional pasture (TP) and 5 days of evaluation. During the first period, one group of animals was assigned to the SPS and the other to the TP; from the second to the fourth period, the groups alternated treatments (Figure 1).

All procedures involving animals were conducted in accordance with protocols approved by the Animal Ethics Committee for Experimental Procedures (CEEA) of the University of Antioquia, under approval number 0146 dated June 7, 2022, ensuring animal welfare.

Table 1. Characteristics of Experimental Animals

Group	Body Weight (kg)	Parity Number	Days in Milk (d)	Milk Yield (kg/cow/day)	Milk fat (%)	Milk protein (%)
1	443 ± 21.35	3.2 ± 2.64	120 ± 32.86	14.2 ± 3.6	4.1 ± 0.07	3.34 ± 0.12
2	462 ± 16.00	3.2 ± 1.60	126 ± 29.39	13.8 ± 1.6	4.0 ± 0.14	3.34 ± 0.14

Two grazing systems were evaluated (Table 2): a silvopastoral system (SPS) consisting of *Piptocoma discolor* planted in double rows spaced 6 m between alleys and 0.5 m between plants (4,200 plants/ha); scattered trees of *Clitoria fairchildiana* and *Guazuma ulmifolia* planted at 20 m × 20 m spacing (25 trees/ha); *Erythrina poeppigiana* used as live fencing with 6 m spacing between individuals (66 trees/ha); and a ground cover of *Urochloa decumbens*. At the time of this study, the SPS had been established for 18 months, and the arboreal species (*C. fairchildiana*, *G. ulmifolia* and *E. poeppigiana*) were still in the early growth stages, without a

developed canopy or tree-like structure. Consequently, the potential impact on microclimate or animal behavior was not considered in the analysis. It was hypothesized that only *P. discolor*, due to its shrubby architecture and high planting density, was capable of contributing structurally to the vegetation stratum.

The traditional pasture system (TP) was based on a monoculture of *Urochloa decumbens*, under extensive grazing with a low tree density (<25 trees/ha), primarily composed of pre-existing *Cordia alliodora* individuals (3–5 years old, 30–40 cm Diameter at Breast Height (DBH)), and managed under a 42-day rotational grazing cycle. While these trees were already established prior to the experiment, their presence reflects the typical structure of low-density arboreal cover in extensively managed tropical pastures, and no additional planting or modification was made for the purpose of this study. All animals received a daily supplement of 3 kg of commercial concentrate throughout the experimental period to ensure a balanced nutritional intake.

Table 2. Nutritional Composition of the Species Evaluated in the Grazing Systems

**Variable**	**Silvopastoral System (SPS)**			**Traditional Pasture (TP)**	
	**Supplement**	***P. discolor***	***U. decumbens***	**Supplement**	***U. decumbens***
DM, %	87 ± 2.98	25.70 ± 1.53	23.0 ± 0.98	87 ± 2.98	22.85 ± 0.68
CP, %	16.02 ± 0.52	25.01 ± 0.69	9.79 ± 0.48	16.02 ± 0.52	8.64 ± 0.26
NDF, %	15.56 ± 0.54	50.01 ± 1.50	59.74 ± 3.01	15.56 ± 0.54	69.81 ± 4.10
ADF, %	7.62 ± 0.25	24.72 ± 1.23	31.87 ± 1.83	7.62 ± 0.25	37.08 ± 1.54
NEL, Mcal*	1.89 ± 0.06	1.49 ± 0.06	1.24 ± 0.09	1.89 ± 0.06	1.10 ± 0.05
TT, g/kg^{-1} DM	-	17.28 ± 0.05	0.08 ± 0.06	-	0.08 ± 0.06
Sap, g/kg^{-1} DM	-	23.46 ± 0.07	1.10 ± 0.05	-	1.10 ± 0.05

DM = dry matter; CP = crude protein; NDF = neutral detergent fiber; ADF = acid detergent fiber; NEL = net energy for lactation. TT = Total Tannins; Sap = Saponins. *Adapted from Buxadé (1994): NEL = 0.677 × DE – 0.359; DE = digestible energy, kcal DE.

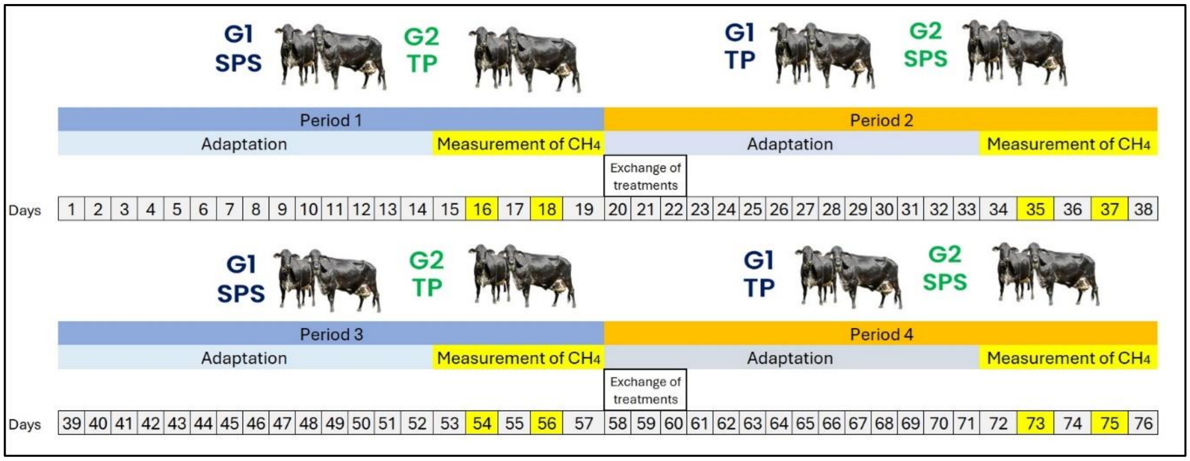

Figure 1. General representation of the experimental design used for sample collection. G1: Group 1 of animals; G2: Group 2 of animals; SPS: silvopastoral system; TP: traditional pasture system.

2.2 Milk yield and dry matter intake (DMI)

Milk yield and DMI data were obtained from a complementary study conducted under the same experimental conditions and using the same animals (Narváez-Herrera et al., 2025). Milk production (kg/day) was determined individually through manual milking performed twice daily. The total volume was measured directly from the calibrated milking bucket immediately after each session. A standard milk density of 1.032 g/mL was used to convert volume to weight. Energy-corrected milk (ECM) was calculated according to Sjaunja et al. (1990), using the following equation:

1) $$ECM\ (kg) = production\ (kg/day)\ * [0.383 * milk\ fat\ \% + 0.242 * milk\ protein\ \% \ + 0.7832) / \ 3.14$$

Milk fat and protein contents were determined by infrared spectroscopy using a MilkoScan FT+ milk analyzer (Foss, Hillerød, Denmark).

Dry matter intake was estimated using two methods, the double marker method and the agronomic method. The double marker combining chromium oxide (Cr_2O_3) as an external marker and acid detergent lignin (ADL) as an internal marker. Cr_2O_3 was administered at a dose of 15 g/cow/day, and fecal samples were collected over five consecutive days per period. The chromium recovery rate was 79.8%. Intake partitioning between forage and supplement was estimated based on differential lignin content, following the procedure described by Mejía-Díaz et al. (2017). A detailed methodological description and the corresponding results are available in Narváez-Herrera et al. (2025). This method was used to estimate the total voluntary intake of forage. The agronomic method was used to determine the grass-to-shrub ratio in the forage diet by measuring the biomass of grasses and shrubs before and after grazing in representative paddocks, following the double sampling technique described by Haydock and Shaw (1975) and adapted by Angulo-Arizala et al. (2022). Measurements were conducted during the pre-experimental period (Figure 1. Adaptation), and the relative proportion of each forage species was used to estimate their contribution to the total voluntary intake. To ensure adequate forage availability and a consistent grass-to-shrub ratio, paddock area and stocking density were adjusted according to animal number and pasture conditions.

2.3 Methane Emission Measurements

Methane measurements were conducted using a laser methane detector (LMD), model LMm-G, manufactured by Crowcon (Erlanger, KY, USA). The device operates based on high-selectivity infrared spectroscopy for the CH_4 absorption band, following the recommendations described by Chagunda et al. (2009, 2013), Ricci et al. (2014), and Lanzoni et al. (2022). Animal measurements were performed by adapting the methodology proposed by Patiño Pardo et al. (2003) for the evaluation of diurnal ingestive behavior in cattle. The behavioral parameters recorded included grazing (PST), ruminating (RM), resting (DE), idle (OC), and milking (OR), avoiding disturbances caused by excessive movement.

Methane was measured in each animal twice daily during two out of the five experimental days: in the morning (06:00–10:30) and in the afternoon (16:30–19:00), during and after the milking routine. To ensure consistency

across measurements, all readings were performed by the same trained operator, who directed the LMD laser beam at the animal's nostril region from a fixed distance of 2 meters. Each measurement lasted for 4 minutes, with data captured every 0.1 seconds, ensuring complete coverage of the eructation and respiration cycles.
During each measurement session, the ambient temperature, relative humidity, and wind speed were recorded using a portable weather station (Davis Vantage Pro2, Hayward, CA, USA). Additionally, in each session, animal behavior and respiration rate were also recorded. To minimize environmental bias, background CH_4 concentration was measured before and after each recording session, ensuring that the values corresponded to enteric emissions.
Methane signal processing was conducted individually for each animal and sampling period, using a dynamic thresholding approach to distinguish baseline respiratory signals from eructation-related peaks (Lanzoni et al., 2022; Pereira et al., 2023). For each measurement profile, the threshold was defined as the arithmetic mean plus one standard deviation of the CH_4 concentration signal. Peaks exceeding this threshold were classified as eructation events, while values below were attributed to baseline respiration. This procedure was applied consistently across all profiles to ensure accurate peak identification across animals and periods. For each animal, the average CH_4 concentration associated with respiration (R_CH_4), eructation (E_CH_4), and the total profile mean (MEAN_CH_4) were calculated. CH_4 concentrations (ppm × m) were subsequently converted to grams of CH_4 per day per kilogram of body weight using the equation proposed by Lanzoni et al. (2022), enabling standardized comparisons across animals.

$$CH_4\ (g\ day^{-1}) = mean\ CH_4\ x\ V\ x\ R\ x\ \propto x\ \beta\ x\ 10^6\ x\ 1440$$

In which V is the tidal volume (3800 mL), R is the respiratory rate (respiratory peaks), ∝ is the conversion factor of CH_4 production from mL to g (0.000667 g/mL), β is the correction factor for the difference between breath and total CH_4 production. The estimation of daily CH_4 emission was normalized to the daily milk production of each cow.
An evaluation protocol was developed to minimise bias in the measurement process of CH4, based on the effect of meteorological data on the quality of measurement with a methane laser detector. Wind direction relative to the position of the laser methane detector (LMD) was classified according to Chagunda et al. (2013) as headwind, crosswind, or tailwind. Field measurements were conducted exclusively when wind speed was less than or equal to 2 m/s, ensuring stable environmental conditions for reliable gas detection. Additionally, the daily Temperature-Humidity Index (THI) was calculated using relative humidity and ambient temperature data, based on the equation proposed by the National Research Council (NRC, 1971):

$$THI = (1.8\ x\ T + 32) - [(0.55 - 0.0055\ x\ HR) x\ (1.8\ x\ T - 26)]$$

2.4 Data processing and statistical analysis

The analysis of the data was conducted using a generalized linear mixed model (GLMM) fitted with the glmmTMB package (Brooks et al., 2017) in R software version 4.3.1 (R Core Team, 2023). A log link function was specified for the response variables (daily methane emission, methane yield, and methane intensity), to correct for their positive skewness and satisfy model assumptions. The model included treatment as a fixed effect and temperature-humidity index (THI) as a continuous covariate. Period, animal group, and individual cow were modeled as random effects.

$$y_{ijkl} = \mu + T_i + \beta + P_j + G_k + C_l + \varepsilon_{ijkl}$$

In which Y is the response variable, μ is the overall mean, T_i is the fixed effect of treatment (i = SPS, TP), β is the regression coefficient for the covariate THI, P_j, G_k, and C_l are the random effects of period (j = 1 to 4), animal group (k = G1, G2), and cow (in-dividual subject), respectively, with $C_l \sim N(0, σ\_c^2)$, and ε_{ijkl} is the residual error term. Model diagnostics were performed using the DHARMa package (Hartig, 2024), based on simulation of scaled residuals. Adjusted means (LSMeans) for the treatment effect were estimated using the emmeans package (Lenth, 2025), and pairwise comparisons were performed using the Tukey test. Statistical significance was declared at $p < 0.05$.

3. Results

3.1 Milk Yield and Dry Matter Intake

Cows managed under the silvopastoral system (SPS) had significantly higher milk yield and dry matter intake (DMI) compared to those in the traditional pasture (TP) (Table 3). These differences were statistically significant ($p < 0.05$) and were used as the basis for calculating methane yield indicators. In the SPS, a grass-to-shrub ratio of 88:12 was observed in the grazed biomass, with an estimated average intake of *Piptocoma discolor* of 1.55 kg DM/animal/day. This value was calculated based on the proportional contribution of the shrub to the total forage biomass, as determined using the agronomic method.

Table 3. Daily dry matter intake (DMI) and milk yield in dual-purpose cows grazing in a silvopastoral system (SPS) and a traditional pasture (TP)

Treatment	DMI (kg/animal/day)	Milk yield (kg/animal/day)
SPS	12.9 ± 0.12	14.13 ± 0.80
TP	10.5 ± 0.12	12.92 ± 0.80
p-value	0.001	0.001

SPS: silvopastoral system; TP: traditional pasture; DMI: dry matter intake.

3.2 Relationship between the Temperature-Humidity Index (THI) and Methane Emissions

The temperature-humidity index (THI) progressively increased across the four evaluation periods, ranging from 75.95 to 87.19 (Table 4). These values were included as a covariate in the statistical model to adjust for environmental variability affecting methane emissions.

Table 4. Temperature-Humidity Index (THI) and daily methane emissions (g CH_4/animal/day) in dual-purpose cows grazing in a silvopastoral system (SPS) and a traditional pasture (TP) across four experimental periods in the Colombian Amazonian foothills.

**Period**	**THI (± SD)**	**SPS CH_4 (g/animal/day)**	**TP CH_4 (g/animal/day)**
1	75.95 ± 1.54	206.13 ± 72.24	269.53 ± 95.87
2	79.85 ± 1.52	222.89 ± 15.03	280.56 ± 44.78
3	81.48 ± 1.47	248.03 ± 26.74	280.04 ± 42.93
4	87.19 ± 1.64	255.10 ± 38.62	278.47 ± 43.78

Values are expressed as mean ± standard deviation. THI: Temperature-Humidity Index. CH_4: methane. SPS: silvopastoral system. TP: traditional pasture.

3.3 Estimation of Methane Emissions Using the LMD

Methane yield indicators reported significant differences between grazing systems (Table 5). Cows in the SPS emitted lower amounts of CH_4 per animal per day, as well as per kilogram of milk, energy-corrected milk (ECM), and dry matter intake (DMI), compared to those in the traditional pasture (TP). All differences were significant ($p<0.05$).

Table 5. Enteric methane emissions (total and intensity-based) in dual-purpose cows grazing a silvopastoral system (SPS) or a traditional pasture (TP) in the Colombian Amazonian foothills.

**Treatment**	**CH_4 (g/animal/day)**	**CH_4 (g/kg milk)**	**CH_4 (g/kg ECM)**	**CH_4 (g/kg DMI)**
SSP	233 ± 6.95 a	15.5 ± 0.356 a	16.0 ± 0.436 a	18.9 ± 0.433 a
TP	277 ± 8.87 b	20.7 ± 0.584 b	21.2 ± 0.659 b	26.7 ± 0.744 b
SEM	6.49215	0.4837	0.3331	0.6085
p-value	0.0001	0.0001	0.0001	0.0001

Values are means ± standard error of the mean (SEM). Different superscript letters in the same column indicate significant differences ($p < 0.05$) according to Tukey’s test. ECM = energy-corrected milk.

3.4 Animal Behavior and Relationship with Methane Emissions

Methane emissions varied according to animal activity in both grazing systems. The highest values were recorded during grazing, followed by resting, and the lowest during ruminating periods (Figure 2). During grazing, cows in the traditional pasture (TP) emitted an average of 321.2 g CH_4/day, which was 5.2% higher than in the silvopastoral system (SPS), where emissions averaged 305.2 g CH_4/day. For resting periods, the TP also showed slightly higher emissions, with an average of 190.2 g CH_4/day compared to 182.4 g CH_4/day in the

SPS, representing an increase of 4.3%. Across all observed activities, methane emissions in the TP were consistently between 1.04 and 1.05 times higher than in the SPS.

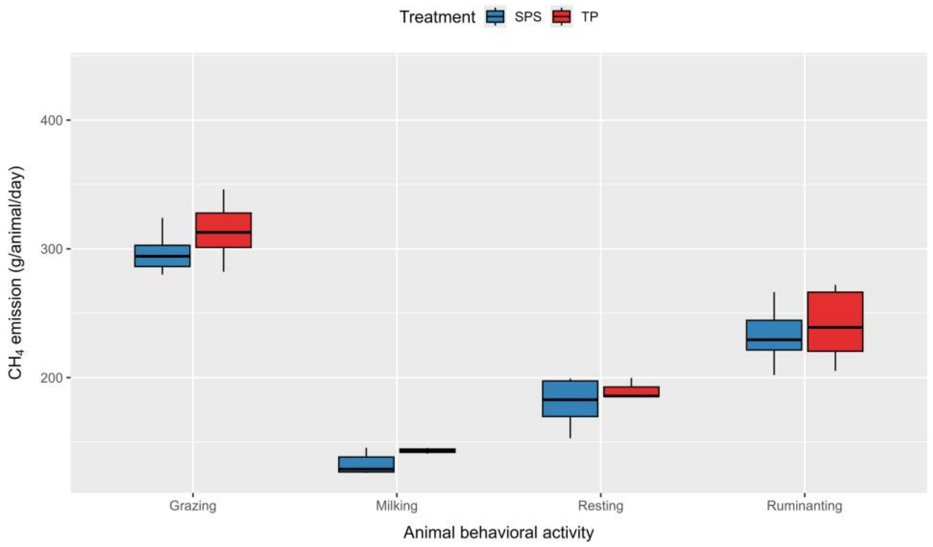

Figure 2. Methane emission patterns according to animal activity

4 Discussion

The quantification of enteric methane emissions using the Laser Methane Detector (LMD) has enabled the generation of representative estimates under real grazing conditions. In our study, the LMD facilitated the differentiation of emissions between two production systems (SPS and TP), reflecting primarily the influence of the feeding regime. Several reports have indicated that the LMD is a useful tool due to its portability, relatively low cost, and capacity to perform multiple non-invasive measurements under field conditions (Sorg et al., 2021). However, technical limitations have also been identified, such as its sensitivity to environmental factors including wind speed, distance, and measurement angle, which can affect the accuracy of the readings (Zhao et al., 2020; Sorg et al., 2021).

Our results support the ability of the LMD to detect differences in CH_4 emissions associated with dietary treatments under grazing conditions. Additionally, emission patterns observed across behavioral activities (grazing, ruminating, milking, and resting) were consistent with previous reports (Arndt et al., 2021), where grazing and ruminating were linked to higher emission peaks due to enhanced rumen fermentation and locomotor activity.

Several studies have demonstrated that diets with greater botanical diversity and improved forage quality can reduce enteric methane production by promoting more efficient rumen fermentation pathways (Jayanegara et al., 2020; Tedeschi et al., 2022). In silvopastoral systems, the inclusion of shrub and tree species with higher digestibility and moderate levels of secondary metabolites such as tannins may shift fermentation toward propionate production, thereby reducing hydrogen availability for methanogenesis (Goel & Makkar, 2012). In

our study, the lower methane emissions observed in the SPS despite higher dry matter intake may be attributed to these compositional and functional differences in the diet. Compared to the traditional pasture, the SPS achieved a reduction of approximately 15.9% in daily CH_4 emissions per animal, suggesting a more efficient use of dietary energy and a potential mitigation benefit under real grazing conditions. The findings of this study were consistent with those of a secondary experiment conducted *in vitro*, which also reported a reduction in methane production with the use of *P. discolor* (Narváez-Herrera et al., 2025).

The findings of the present study are in accordance with those reported by Rivera et al. (2022), who observed emissions ranging from 207 to 228 g CH_4/animal/day in pasture-based systems with *Brachiaria humidicola* and 15% inclusion of *Tithonia diversifolia*, using polytunnels for *in vivo* quantification. Similarly, Eismann et al. (2024) reported 205 g CH_4/animal/day in Jersey cows under European pasture systems, using the eddy covariance (EC) technique, which employs remote sensors to quantify trace gas fluxes between the surface and the atmosphere. On the other hand, using the LMD and a respiratory-based equation incorporating tidal volume, respiratory rate, and standard conversion factors (Chagunda et al., 2009), Lanzoni et al. (2022) reported emissions of 328.6 ± 160.0 g CH_4/animal/day in Mediterranean buffaloes. Likewise, Pereira et al. (2023) using the LMD under controlled conditions and a 120-second sampling duration, reported 53.9 g CH_4/animal/day in Jersey cows and 60.7 g CH_4/animal/day in Holstein cows. According to Boré et al. (2022), although the LMD is sensitive to sampling duration, it yields reproducible estimates when measurement conditions are standardized. A minimum of 240 seconds of measurement is recommended to significantly improve precision, a criterion that was met in the present study, where at least 240 seconds were used per animal per measurement event. This approach may contribute to the consistency of the data obtained. However, as noted by Sorg (2021), the lack of a standardized protocol for LMD-based measurements and data analysis still limits comparability across studies and the overall validity of methane production estimates.

Beyond absolute emissions, methane intensity is widely regarded as a more robust indicator of environmental efficiency, as it accounts for the animal's productive output (de Haas et al., 2021). In our study, cows managed under the silvopastoral system (SPS) showed significantly lower CH_4 intensities per kilogram of milk, energy-corrected milk (ECM), and dry matter intake (DMI), reflecting a more efficient use of ingested nutrients. Specifically, methane yield per kilogram of DMI in the SPS was reduced by approximately 29% compared to the traditional pasture (TP), and CH_4 per kilogram of ECM was 25% lower, highlighting better feed conversion efficiency. These improvements are likely linked to the superior nutritional quality of the forage base in the SPS, which included *Piptocoma discolor*, a specie characterized by lower fiber content, higher crude protein (up to 27.5%), and greater energy availability (up to 1.52 Mcal/kg DM), particularly during early regrowth stages in the rainy season (unpublished data). This species also contains functional secondary metabolites such as tannins and saponins, which may modulate ruminal fermentation and improve nitrogen utilization efficiency. Similar variability in methane intensity across systems and metrics has been reported by Eismann et al. (2024), with values ranging from 5.4 to 12.47 g CH_4/kg ECM in Jersey cows under supplemented grazing conditions. The higher intensity values observed in our study are consistent with animals under full grazing and limited

concentrate input. The overall differences between systems likely reflect the lower nutritional quality and higher fiber content (particularly ADF) of *Urochloa decumbens* in the TP, in contrast to the higher-quality forage base in the SPS, including *Piptocoma discolor*.

Comparatively, Loza et al. (2021) reported 12.3 g CH_4/kg DMI in intensive systems with high supplementation, and Pereira et al. (2023) found even lower values (11.1 g CH_4/kg DMI) in Holstein cows under rotational grazing with concentrate. Similarly, Noguera and Posada (2017) reported 16.1 g CH_4/kg DMI and 11.9 g CH_4/kg milk in high-yielding Holstein cows (27 kg milk/day) housed under a fully confined system with Kikuyo grass and concentrate fed according to milk yield. These values reflect the impact of concentrate inclusion and controlled intake on fermentation efficiency. In contrast, the higher methane yield observed in the traditional pasture (TP) in our study (26.7 g CH_4/kg DMI and 20.7 g CH_4/kg milk) suggests that the base forage, dominated by *Urochloa decumbens*, has lower digestibility and fiber quality. Conversely, the silvopastoral system (SPS) exhibited a more efficient profile (18.9 g CH_4/kg DMI; 15.5 g CH_4/kg milk), possibly due to greater forage diversity and the inclusion of species such as *Piptocoma discolor*, which may enhance ruminal fermentation. Rivera et al. (2022) observed similar improvements when incorporating *Tithonia diversifolia* into tropical diets, reinforcing the role of plant functional traits in methane mitigation strategies.

When considered collectively, methane intensity and yield indicators suggest that SPS improves the environmental efficiency of dual-purpose production systems not only by reducing total emissions but also by decreasing methane output per unit of milk produced and per kilogram of dry matter intake. This finding is consistent with the report by Sierra-Alarcón et al. (2022), who found that Holstein cows in a silvopastoral system emitted 246.7 g CH_4/animal/day, representing a 15.4% reduction compared to the 291.5 g CH_4/animal/day recorded in the traditional pasture system. Moreover, lower emission intensities were observed per unit of output: 13.7 g CH_4/kg-1 fat-corrected milk (FCM) in the SPS versus 23.8 g CH_4/kg-1 FCM in the traditional system, and 14.1 vs. 18.6 g CH_4/kg-1 DMI, respectively. The inclusion of tree and shrub species such as *Eucalyptus* sp., *Alnus acuminata*, *Acacia melanoxylon*, and *Sambucus peruviana* improved the nutritional quality of the diet, increased FCM yield (19.1 vs. 12.3 kg FCM/day-1), and reduced the Ym factor (3.4% vs. 4.5%), an indicator of energy losses as methane. These results demonstrate greater energy efficiency in the SPS and reinforce its potential as an integrated strategy to mitigate emissions in tropical livestock systems.

Enteric methane emissions in cattle are closely linked to behavioral activity, as variations in intake rate, fermentation dynamics, and digestive efficiency influence CH_4 output (Olijhoek et al., 2022). As reported by Watt et al. (2015), cows with longer rumination times emitted 18% more CH_4 per day, partly due to increased feed intake. From a fermentative standpoint, higher intake of forages rich in acid detergent fiber (ADF) such as *Urochloa decumbens* promotes the activity of cellulolytic bacteria, which preferentially degrade structural carbohydrates. This degradation favors acetate production, associated with elevated hydrogen release, thereby stimulating methanogenesis (Wang et al., 2020; Cuervo et al., 2025). In contrast, the silvopastoral system (SPS) included shrub species with lower ADF content, higher digestibility, and the presence of secondary metabolites

such as condensed tannins, which can suppress methanogenic archaea or modulate microbial fermentation (Goel & Makkar, 2012; Cuervo et al., 2025). These attributes contribute to a more efficient fermentation profile with reduced hydrogen availability, helping to explain the lower methane yield observed in the SPS, even under higher dry matter intake.

In our experiment, higher emission rates were observed during grazing, particularly in the TP system, where CH_4 output exceeded that of the SPS by approximately 13.5%. This finding is consistent with the conclusions of earlier studies, which indicated that active forage intake stimulates fermentative hydrogen (H_2) production. This process is the primary substrate used by methanogenic archaea to reduce carbon dioxide (CO_2) into methane (CH4) (Hristov et al., 2013; Kang et al., 2022). Moreover, grazing often coincides with periods of elevated thermal load during the day, which may increase respiratory rate and pulmonary ventilation. As a consequence, the frequency of methane exhalation may rise due to enhanced tidal volume and intensified gas exchange, as observed under field conditions with spirometric monitoring systems (Correa-Cardona & Jaimes-Cruz, 2023). Methane emissions during rumination showed greater variability, this is likely due to the release of accumulated gases via eructation, a process closely linked to the secondary contraction cycle of the reticulorumen (Roessler & Schlecht, 2021; Sorg, 2022). While primary fermentation activity is reduced during rumination, measurable CH_4 peaks can still be detected, likely reflecting residual fermentative activity and eructation of previously accumulated gases (Brask et al., 2015).

On the other hand, when animals were at rest, emissions decreased notably, which can be attributed to a lower level of ruminal fermentation and reduced digestive metabolism. The relationship between emission and activity observed in the present study was similar to Marçal-Pedroza et al. (2021), who reported that cows exhibiting lower activity levels inside respiration chambers characterized by longer lying times and fewer steps had lower CH_4 emission rates, greater energy efficiency, and lower CH_4 energy losses per liter of milk. In this context, passive behavior was a predictor of lower emission intensity, highlighting the importance of recording behavioral state during gas quantification. Likewise, Smith et al. (2024) reported that animals at rest, which did not voluntarily approach the GreenFeed system, exhibited significantly lower CH_4 emission rates, as measurement in that system depends on the animal's active access during feeding. These findings support the assertion that even during resting states, baseline emissions from pulmonary ventilation should be accounted for (Sorg, 2022).

During the milking routine, CH_4 emissions were minimal. This activity represents a phase of low digestive activity, in which neither intake nor rumination occurs, and where human interaction can influence respiratory patterns (Marçal-Pedroza et al., 2021). Some studies suggest that during milking or brief confinement, the respiratory pattern may become more irregular but does not significantly increase CH_4 concentration (Roessler et al., 2021; Kang et al., 2022). Additionally, animals in the SPS showed lower emissions across all recorded activities, which may be attributed primarily to the compositional quality of the diet, characterized by lower fiber content and higher digestibility. Although the trees in the experimental setup were still under development

and did not yet provide full canopy cover, it is plausible that even partial structural vegetation and the diversified environment may have contributed to microclimatic buffering and improved animal behavior, as observed in more established silvopastoral systems (Muñoz et al., 2021; Orlindo et al., 2022). However, further research is needed to isolate and quantify the specific contribution of tree cover once full canopy development is achieved. Furthermore, the use of the LMD in behavioral studies represents a valuable tool to identify temporal and physio-logical patterns of CH_4 emission. However, its accuracy depends critically on the standardization of the measurement protocol, as emphasized in recent methodological studies (Boré et al., 2022; Sorg, 2022).

5 Conclusions

The implementation of silvopastoral systems (SPS) with promising shrub and tree species in the Colombian Amazonian foothills demonstrated clear advantages by reducing enteric methane emissions (by up to 25% per kilogram of milk) and improving the environmental efficiency of dual-purpose cattle production. These effects are closely linked to enhanced diet quality, including increased crude protein, digestibility, and the presence of functional secondary metabolites such as tannins and saponins. The use of the Laser Methane Detector (LMD), although requiring strict field protocols, proved to be a practical and non-invasive tool for detecting emission patterns associated with animal behavior in tropical grazing systems. Taken together, these findings highlight the potential of SPS as a comprehensive strategy for mitigating greenhouse gas emissions in livestock production and emphasize the need for integrated efforts in research, extension, and public policy to scale up its adoption in tropical regions.

Declaration

Funding: To achieve the results of this research, various technical and financial funding sources were sought: The Ministry of Science, Technology, and Innovation of Colombia, the Bicentennial Excellence Doctoral Scholarship Program - Cohort II, Doctoral Scholarship Agreement No. 20230030-20-21, and doctoral study funds for Juan Pablo Narváez-Herrera. Additionally, this research received technical support from the GRICA Research Group – Sustainable Animal Production Systems Line at the University of Antioquia, as well as the National Learning Service (SENA) – Putumayo Regional Office.

Conflict of Interest: The authors declare no conflicts of interest. The funding sources was not involved in the study design; data collection, analysis, or interpretation; manuscript writing; or the decision to publish the results.

Ethical Approval: The procedures described in this study were conducted following protocols approved by the Ethics Committee for Animal Experimentation (CEEA) at the University of Antioquia under Act No. 0146 of June 7, 2022, ensuring animal welfare. This manuscript does not include clinical studies or patient data.

Consent to Participate: Not applicable.

Consent for Publication: Not applicable.

Abbreviations

CH_4	Methane
CO_2	Carbon Dioxide
CP	Crude Protein
DE	Digestible Energy
DMI	Dry Matter Intake
ECM	Energy Corrected Milk
ENL	Net Energy for Lactation
ADF	Acid Detergent Fiber
NDF	Neutral Detergent Fiber
GHG	Greenhouse Gas
THI	Temperature-Humidity Index
LMD	Laser Methane Detector
DM	Dry Matter
MY	Milk Yield
SPS	Silvopastoral System
TP	Traditional Pasture

References

Angulo-Arizala, J., Nemocón-Cobos, A., Barragán-Hernández, W. A., Gallo-Marín, J., & Mahecha-Ledesma, L. (2022). Food industry residues (snacks) as feed in a dairy farm in the Colombian high tropics. *Ciencia y Tecnología Agropecuaria*, *23*(1), e2055. https://doi.org/10.21930/rcta.vol23_num1_art:2055

Arndt C., Price W.J., Pelaez A.M., Oh J., Bayat A.R., Dijkstra J., Kebreab E., McGee M., Newbold C.J., Schwarm A., et al. (2021) Strategies to mitigate enteric methane emissions by ruminants—A way to approach the 2.0 °C target. agriRxiv. https://doi.org/10.31220/agriRxiv.2021.00040

Azevedo, A. R., Lopes, M. S., Borba, A., da Câmara Machado, A., & Mendonça, D. (2024). *Exploring the Catrina, an autochthonous cattle breed of the Azores, for a comparative analysis of methane emissions with Holstein-Friesian dairy cows. Frontiers in Animal Science*, *5*, 1423940. https://doi.org/10.3389/fanim.2024.1423940

Brask, M., Weisbjerg, M. R., Hellwing, A. L. F., Bannink, A., & Lund, P. (2015). Methane production and diurnal variation measured in dairy cows and predicted from fermentation pattern and nutrient or carbon flow. *Animal*, *9*(11), 1795–1806. https://doi.org/10.1017/S1751731115001184

Boré, R., et al. (2022). Measurement duration but not distance, angle, and neighbour-proximity affects precision in enteric methane emissions... Animals, 12(1295). https://doi.org/10.3390/ani12101295

Brooks, M. E., Kristensen, K., van Benthem, K. J., Magnusson, Á., Berg, C. W., Nielsen, A., Skaug, H. J., Mächler, M., & Bolker, B. M. (2017). glmmTMB balances speed and flexibility among packages for

zero-inflated generalized linear mixed modeling. *The R Journal*, *9*(2), 378–400. https://doi.org/10.32614/RJ-2017-066

Buxadé C (1995) Zootecnia. Bases de producción animal. Tomo II. Reproducción y alimentación. Madrid (España): Mundi-prensa, 344 p.

Correa Cardona, H. J., & Jaimes Cruz, L. J. (2023). Design and operation of a spirometry mask to quantify exhaled methane emission by grazing cattle. *Livestock Research for Rural Development*, 35(9), Volume 35, Article #83. http://www.lrrd.org/lrrd35/9/3583hjco.html

Chagunda, M. G. G., Ross, D., & Roberts, D. J. (2009). On the use of a laser methane detector in dairy cows. Computers and Electronics in Agriculture, 68(2), 157–160. https://doi.org/10.1016/j.compag.2009.05.008

Chagunda, M. G. G., Ross, D., Rooke, J., Yan, T., Douglas, J. L., Poret, L., McEwan, N. R., Teeranavattanakul, P., & Roberts, D. J. (2013). Measurement of enteric methane from ruminants using a hand-held laser methane detector. Acta Agriculturae Scandinavica, Section A — Animal Science, 63(2), 68–75. https://doi.org/10.1080/09064702.2013.797487

Cuervo, W., Gomez-Lopez, C., & DiLorenzo, N. (2025). Methane Synthesis as a Source of Energy Loss Impacting Microbial Protein Synthesis in Beef Cattle—A Review. *Methane*, *4*(2), 10. https://doi.org/10.3390/methane4020010

de Haas, Y., Veerkamp, R. F., de Jong, G., & Aldridge, M. N. (2021). Selective breeding as a mitigation tool for methane emissions from dairy cattle. *Animal: an international journal of animal bioscience*, *15 Suppl 1*, 100294. https://doi.org/10.1016/j.animal.2021.100294

Delagarde, R., Guyard, R., Boré, R., & Rouillé, B. (2024). *Strategies of maize silage supplementation of grazing dairy cows: Effects on milk production, pasture intake, grazing behaviour and methane emissions*. *Data in Brief*, *54*, 110361. https://doi.org/10.1016/j.dib.2024.110361

Eismann, M.-S. R., Smit, H. P. J., Poyda, A., Loges, R., Kluß, C., & Taube, F. (2024). *Combining the Eddy Covariance Method and Dry Matter Intake Measurements for Enteric Methane Emission Estimation from Grazing Dairy Cows*. *Atmosphere*, *15*(1269). https://doi.org/10.3390/atmos15111269

Goel, G., & Makkar, H. P. (2012). Methane mitigation from ruminants using tannins and saponins. *Tropical animal health and production*, *44*(4), 729–739. https://doi.org/10.1007/s11250-011-9966-2

Grainger, C., Clarke, T., McGinn, S. M., Auldist, M. J., Beauchemin, K. A., Hannah, M. C., Waghorn, G. C., Clark, H., & Eckard, R. J. (2007). Methane emissions from dairy cows measured using the sulfur hexafluoride (SF6) tracer and chamber techniques. *Journal of Dairy Science*, *90*(6), 2755–2766. https://doi.org/10.3168/jds.2006-697

Hai, T., Liu, J., Wang, H., & Zhang, S. (2025). *Loss of Myostatin leads to low production of CH4 by altering rumen microbiome and fermentation pattern in cattle*. *Journal of Animal Science*, *108*(2), 322–355. https://doi.org/10.3168/jds.2024-25050

Hammond, K. J., Crompton, L. A., Bannink, A., Dijkstra, J., Yáñez-Ruiz, D. R., O'Kiely, P., Kebreab, E., Eugène, M., Yu, Z., Shingfield, K. J., Schwarm, A., Haugen, J. E., & Reynolds, C. K. (2016). *Review of current in vivo measurement techniques for quantifying enteric methane emission from ruminants*. *Animal Feed Science and Technology*, *219*, 13-30. https://doi.org/10.1016/j.anifeedsci.2016.05.018

Hammond, K. J., Humphries, D. J., Crompton, L. A., Green, C., & Reynolds, C. K. (2015). Methane emissions from cattle: Estimates from short-term measurements using a GreenFeed system compared with measurements obtained using respiration chambers or sulphur hexafluoride tracer. *Animal Feed Science and Technology*, *203*, 41–52. https://doi.org/10.1016/j.anifeedsci.2015.02.008

Hartig, F. (2024). DHARMa*: Residual diagnostics for hierarchical (multi-level / mixed) regression models* (R package version 0.4.7). http://florianhartig.github.io/DHARMa/

Haydock, K. P., & Shaw, N. H. (1975). The comparative yield method for estimating dry matter yield of pasture. *Australian Journal of Experimental Agriculture and Animal Husbandry*, *15*, 663–670. https://doi.org/10.1071/EA9750663

Holdridge LR (1966) "The life zone system." Adansonia 6.2: 199-203.

Hristov, A. N., et al. (2013). Mitigation of greenhouse gas emissions in livestock production – A review. FAO Animal Production and Health Paper No. 177.

IGAC – Instituto Geográfico Agustín Codazzi. 2024. Soil classification in tropical ecosystems: Ultisols in the Amazon. Colombian Geographical Institute Bulletin; 12(3); 45-57.

Intergovernmental Panel on Climate Change (IPCC). (2021). Climate Change 2021: The Physical Science Basis. Contribution of Working Group I to the Sixth Assessment Report of the IPCC. Cambridge University Press. https://www.ipcc.ch/report/ar6/wg1/

Jayanegara, A., Yogianto, Y., Wina, E., Sudarman, A., Kondo, M., Obitsu, T., & Kreuzer, M. (2020). Combination Effects of Plant Extracts Rich in Tannins and Saponins as Feed Additives for Mitigating in Vitro Ruminal Methane and Ammonia Formation. *Animals*, *10*(9), 1531. https://doi.org/10.3390/ani10091531

Kang, K., et al. (2022). Application of a hand-held laser methane detector for measuring enteric methane emissions... Journal of Animal Science. https://doi.org/10.1093/jas/skac211

Lanzoni, L., Chagunda, M. G. G., Fusaro, I., Chincarini, M., Giammarco, M., Atzori, A. S., Podaliri, M., & Vignola, G. (2022). Assessment of Seasonal Variation in Methane Emissions of Mediterranean Buffaloes Using a Laser Methane Detector. *Animals*, *12*(24), 3487. https://doi.org/10.3390/ani12243487

Lenth R (2025). emmeans*: Estimated Marginal Means, aka Least-Squares Means*. R package version 1.11.1-00001, https://github.com/rvlenth/emmeans.

Loza, C.; Reinsch, T.; Loges, R.; Taube, F.; Gere, J.I.; Kluß, C.; Hasler, M.; Malisch, C.S. (2021). Methane Emission and Milk Production from Jersey Cows Grazing Perennial Ryegrass–White Clover and Multispecies Forage Mixtures. *Agriculture*, *11*, 175. https://doi.org/10.3390/agriculture11020175

Ma, W., Ji, X., Ding, L., Yang, S. X., Guo, K., & Li, Q. (2024). *Automatic Monitoring Methods for Greenhouse and Hazardous Gases Emitted from Ruminant Production Systems: A Review*. *Sensors, 24*(4423). https://doi.org/10.3390/s24134423

Marçal-Pedroza, M. G., Campos, M. M., Sacramento, J. P., Pereira, L. G. R., Machado, F. S., Tomich, T. R., Paranhos da Costa, M. J. R., & Sant'Anna, A. C. (2021). Are dairy cows with a more reactive temperament less efficient in energetic metabolism and do they produce more enteric methane? Animal, 15, 100224. https://doi.org/10.1016/j.animal.2021.100224

Mejia-Diaz E, Mahecha-Ledesma L, Angulo-Arizala J (2017) Dry matter intake in a silvopastoral system of *T. diversifolia* in high tropics. *Agronomía Mesoamericana*, 28(2), 389–403. https://doi.org/10.15517/ma.v28i2.23561

Meo Zilio, D., Iacurto, M., Cenci, F., & Steri, R. (2024). *Methane Emission of Italian Mediterranean Buffaloes Measured Using a Laser Detector During a Lactation Cycle*. *Animals*, *14*(3652). https://doi.org/10.3390/ani14243652

Monteiro, A., Alves, S. P., Bessa, R. J. B., & Jerónimo, E. (2024). *Forestry Systems as a Strategy for Mitigating Greenhouse Gas Emissions in Livestock Production: A Review*. *Sustainability*, *16*(3), 2219. https://doi.org/10.3390/su16052219

Muñoz, C., et al. (2021). Long-term and carryover effects of supplementation with whole oilseeds... Animals, 11(2978). https://doi.org/10.3390/ani11102978

Narváez-Herrera JP, Angulo-Arizala J, Barragán-Hernández W, Mahecha-Ledesma L. Chemical composition and in vitro ruminal methane production of native fodder trees and shrubs in the Amazonian piedmont. *Rev Bras Zootec*. In press. 2025.

Narváez-Herrera JP, Angulo-Arizala J, Barragán-Hernández WA, Riascos-Guerrero YM, Mahecha-Ledesma L. From leaves to liters: Enhancing dairy efficiency through silvopastoral integration in the Amazonian foothills. *Manuscript submitted for publication*. 2025.

Noguera, R. R., & Posada, S. L. (2017). Enteric methane emission factor for lactating Holstein cows in northern Antioquia, Colombia. *Livestock Research for Rural Development, 29*, Article #119. http://www.lrrd.org/lrrd29/6/ricn29119.html

Nunes, H. P. B., Maduro Dias, C. S. A. M., Vouzela, C. M., & Borba, A. E. S. (2024). *Seasonal Effect of Grass Nutritional Value on Enteric Methane Emission in Islands Pasture Systems*. *Animals, 13*(2766). https://doi.org/10.3390/ani13172766

Olijhoek, D. W., Hellwing, A. L. F., Noel, S. J., Lund, P., Larsen, M., Weisbjerg, M. R., & Børsting, C. F. (2022). Feeding up to 91% concentrate to Holstein and Jersey dairy cows: Effects on enteric methane emission, rumen fermentation and bacterial community, digestibility, production, and feeding behavior. *Journal of Dairy Science, 105*(12), 9523–9541. https://doi.org/10.3168/jds.2021-21676

Orlindo, L. T., et al. (2022). Quantification of methane emitted by ruminants: A review of methods. Journal of Animal Science. https://doi.org/10.1093/jas/skac197

Patiño Pardo, R. M., Fischer, V., Balbinotti, M., Moreno, C. B., Ferreira, R. E. X., Vinhas, I., & Monks, P. L. (2003). Comportamento ingestivo diurno de novilhos em pastejo submetidos a níveis crescentes de suplementação energética [Diurnal ingestive behavior of grazing steers fed increasing levels of energy supplementation]. *Revista Brasileira de Zootecnia, 32*(6), 1408–1418. https://doi.org/10.1590/S1516-35982003000600016

Pereira, J. C., Silva, J. D., Souza, L. R., & Albuquerque, L. G. (2023). *Genetic Analysis of Methane Emission Traits in Holstein Cattle Using a Laser Methane Detector (LMD)*. *Journal of Dairy Science, 106*(4), 2541–2553. https://doi.org/10.3168/jds.2022-22845

Ricci, P., Chagunda, M. G. G., Rooke, J., Houdijk, J. G. M., Duthie, C. A., Hyslop, J., Roehe, R., & Waterhouse, A. (2014). Evaluation of the laser methane detector to estimate methane emissions from ewes and steers. *Journal of Animal Science, 92*(11), 5239–5250. https://doi.org/10.2527/jas.2014-7676

Rivera JE, Villegas G, Chará J, Durango SG, Romero MA, Verchot L (2022) Effect of *Tithonia diversifolia* intake on in vivo methane (CH4) emission and milk production in dual-purpose cows in the Colombian Amazonian piedmont. *Translational Animal Science, 6*(1), 1-12. https://doi.org/10.1093/tas/txac139

Roessler, R., & Schlecht, E. (2021). Application of the laser methane detector for measurements in freely grazing goats... Animal, 15, 100070. https://doi.org/10.1016/j.animal.2020.100070

Sierra-Alarcón, A. M., Adegbeye, M. J., Benavides, J. C., Mayorga, O., et al. (2022). Enteric methane from dairy cattle grazing on traditional pastoral and silvopastoral systems of Cundinamarca farms [Poster presentation]. 8th International Greenhouse Gas & Animal Agriculture Conference. https://doi.org/10.13140/RG.2.2.27728.79365

Sjaunja, L. O., Junkkarinen, L., Pedersen, J., & Setälä, J. (1990). A Nordic proposal for an energy corrected milk (ECM) formula. In P. Gaillon & Y. Chabert (Eds.), Performance recording of animals: State of

the art. 27th biennial session of the International Committee for Animal Recording (pp. 156–192). Centre for Agricultural Publishing and Documentation. https://eurekamag.com/research/002/007/002007239.php

Smith, O. A., Rochus, C. M., Baes, C. F., & van Staaveren, N. (2024). A note on dairy cow behavior when measuring enteric methane emissions with the GreenFeed emission monitoring system in tiestalls. JDS Communications, 5, 395–399. https://doi.org/10.3168/jdsc.2023-0451

Sorg, D. (2022). Measuring livestock CH_4 emissions with the laser methane detector: A review. Methane, 1(1), 38–57. https://doi.org/10.3390/methane1010004

Tedeschi LO, Abdalla AL, Álvarez C, Anuga SW, Arango J, Beauchemin KA, Becquet P, Berndt A, Burns R, De Camillis C, Chará J, Echazarreta JM, Hassouna M, Kenny D, Mathot M, Mauricio RM, McClelland SC, Niu M, Onyango AA, Parajuli R, Pereira LGR, Del Prado A, Paz Tieri M, Uwizeye A, Kebreab E. (2022). Quantification of methane emitted by ruminants: a review of methods. J Anim Sci. Volume 100, Issue 7, https://doi.org/10.1093/jas/skac197

Wang, L., Zhang, G., Li, Y., & Zhang, Y. (2020). Effects of High Forage/Concentrate Diet on Volatile Fatty Acid Production and the Microorganisms Involved in VFA Production in Cow Rumen. *Animals*, *10*(2), 223. https://doi.org/10.3390/ani10020223

Watt, L. J., Clark, C. E. F., Krebs, G. L., Petzel, C. E., Nielsen, S., & Utsumi, S. A. (2015). Differential rumination, intake, and enteric methane production of dairy cows in a pasture-based automatic milking system. *Journal of Dairy Science*, *98*(10), 7248–7263. https://doi.org/10.3168/jds.2015-9463

Zhao, Y., Nan, X., Yang, L., Zheng, S., Jiang, L., & Xiong, B. (2020). A Review of Enteric Methane Emission Measurement Techniques in Ruminants. *Animals*, *10*(6), 1004. https://doi.org/10.3390/ani10061004

Total number of words (including references): 7762
Total number of words (excluding references): 5714

Abstract number of words: 203

Number of words in Supplementary Information: (Total: 0)

Supplementary material 1: 0

Supplementary material 2: 0

Supplementary material 3: 0
