# Supplementary material for: Estimation of enteric methane emissions in dairy cows under grazing a silvopastoral system and a grass monoculture in the Colombian Amazonian foothills
Source: PLoS One. 2026 Jan 30;21(1):e0337719. doi: 10.1371/journal.pone.0337719 (PMC12857995; doi:10.1371/journal.pone.0337719)
Supplement: Attachment — Submitted filename: Response to reviewers 151025.pdf [file pone.0337719.s006.pdf]

Medellín, Colombia 15-10-2025

Dear Editor and Reviewers PLOS ONE Journal

Subject: Manuscript Revision - **ID:** PONE-D-25-33619

Title: *Estimation of enteric methane emissions in dairy cows under grazing a silvopastoral system and a grass monoculture in the Colombian Amazonian Foothills*

We sincerely thank you for the thorough evaluation and constructive feedback. We have revised the manuscript extensively to address all points. Below we provide a detailed, point-by-point response indicating what changed and where (section/table/figure), and quoting newly added or modified text when appropriate. We submit: (i) a revised manuscript with Track Changes, (ii) a clean version, and (iii) this response letter.

Review Report

Abstract

1) Please clearly state the research area and articulate the study's novelty and relevance.
Response. We revised the opening sentences to explicitly situate the study in humid tropical grazing systems and highlight the novel field application of LMD under Colombian Amazon foothill conditions, emphasizing implications for mitigation metrics and inventories.
Changes. Abstract, lines 1–6: Added "humid tropics," "field-based," and "inventory relevance."

2) Why focus on LMD rather than other methods?
Response. We added a sentence justifying LMD: non-invasive, portable, suitable for repeated measures under grazing, and complementary to chambers/SF_6 where logistics limit deployment.

3) Were animals classified by feed type or activity? Why?
Response. We clarify that emissions were analyzed by animal activity state (grazing, ruminating, resting, milking) to link behavior with flux patterns; treatment differences (SPS vs. TP) are presented separately.

4) Add limitations/strengths.
Response. We now summarize limitations (sample size, short window, lack of 24-h coverage/standardization) and strengths (field realism, activity-resolved profiles).

Introduction

5) Background useful but lacks cohesion; some paragraphs lack or use old citations.
Response. We updated and expanded citations (2020–2024) on enteric CH_4 fundamentals and removed outdated placeholders. We replaced the 1966 Holdridge reference with the Köppen-Geiger climate classification (Kottek et al., 2006) already cited in Methods.

6) Other studies using LMD not indicated.
Response. We added a concise paragraph summarizing LMD validation and use under housed and grazing conditions (e.g., Chagunda 2009, 2013; Ricci 2014; Lanzoni 2022; Pereira 2023; Meo Zilio 2024; Nunes 2024) and noted sources of variability.

7) Advantage/disadvantage of each production system (feed resource, management).
Response. We now explicitly contrast SPS (diet quality/diversity, microclimate; higher establishment/labor) with TP (lower CP, higher fiber, seasonal bottlenecks).

Materials and Methods

8) Clarify rationale behind experimental design and sampling periods.
Response. We state that a 4-period two-sequence crossover allowed each cow to serve as its own control and helped mitigate carry-over; we justify 14-day adaptation (per Machado 2016; Grainger 2007) and 5-day measurement (per Hammond 2016).

9) Use recent literature (olds citation 1966).
Response. As above, we removed the Holdridge (1966) life-zone citation and retained Köppen-Geiger (Kottek 2006) in Study Site.
Changes. Section 2.1 (Study site): updated climate description.

10) DMI/forage intake calculation unclear.
Response. We provide a succinct description of the double-marker approach (Cr_2O_3/ADL), chromium recovery, and the agronomic double-sampling method to partition grass vs. shrub contributions; we also specify the forage offer target (10–12% BW as kg $DM \cdot cow^{-1} \cdot day^{-1}$) used to adjust paddock area/stocking through periods.

11) Behavioral observations not fully defined (duration, frequency, observers).
Response. We added the instantaneous scan sampling every 2 min within the 4-min LMD windows, two trained observers, inter-observer agreement (>90%), and blinding to treatment during scoring.

12) Sampling days—selection criteria?
Response. We specify that two of the five measurement days per period were selected based on a priori environmental thresholds (wind $\leq 2\ m \cdot s^{-1}$, no rain, stable conditions) to ensure LMD data quality.

13) THI not in Methods.
Response. THI computation (NRC, 1971), variables, and device are now fully described.
Changes. Section 2.5 (THI): added formula and instrumentation.

14) Statistical model clarity (fixed effects).
Response. We clarify that treatment (SPS vs. TP) is the sole fixed effect of interest; THI is a covariate; period, group, and cow are random effects. No activity-specific GLMMs were run; activity effects are descriptive.

Results

15) Clarify diet species counted in SPS vs. TP.
Response. We added a note under the forage composition table indicating that tree species present in SPS contributed negligibly to intake during the study and were not included in DMI partitioning.

16) Include n and statistics in tables; standardize expressions; fix SEM labels.
Response. We now report mean ± SEM, sample size (n = 10; 5 per sequence per period), and GLMM p-values. Column superscripts and footnotes specify Tukey post-hoc tests ($p < 0.05$).

17) Figure does not show significance.

Response. Figure 2 caption now indicates significant differences via letter superscripts (Tukey, $p < 0.05$). Changes. Figure 2 legend updated.

Discussion

18) Elevate cohesion and integrate limitations/standardization.
Response. We front-load a paragraph explaining the LMD protocol safeguards (2 m distance; ≥240 s; wind ≤ 2 $m \cdot s^{-1}$), emphasize within-study contrasts, and acknowledge limits for cross-study quantitative comparisons.

19) Explain how LMD accuracy/limitations were handled after listing them.
Response. As above; explicit link between protocol and data quality added.

20) Relate diet mechanisms to observed mitigation; avoid redundancy.
Response. We merged overlapping paragraphs to state: SPS reduced CH_4 despite higher DMI, consistent with diet quality (incl. Piptocoma discolor and functional metabolites) shifting fermentation/limiting H_2 availability; then link to lower CH_4 per kg DMI and higher milk yield (dilution of maintenance).

Conclusions

21) Relate conclusions to objectives, include limitations, avoid policy 'recommendations' as conclusions.
Response. Conclusions now: (i) restate the SPS vs. TP mitigation finding across intensity metrics; (ii) explicitly tie to diet quality and activity-resolved LMD signals; (iii) list key limitations (sample, duration, LMD standardization); (iv) avoid prescriptive policy language while noting relevance to inventories.

Grammar, Consistency, and References

22) Grammar/consistency/proofreading issues.
Response. We completed a line-by-line language edit for tense consistency, unit formatting (SI), and term standardization.

23) Proper citation and referencing.
Response. All in-text citations now match the reference list, which is formatted per PLOS ONE numeric style (Vancouver-like; journal name unabbreviated; DOI where available).

24) Lack order 108, 113.
Response. We corrected the numbering/ordering issue flagged (formerly duplicated or misordered items around those positions).

Editorial Compliance (for transparency)
Figure 1: Figure 1 was eliminated, the design used and the treatment scheme in materials and methods are described in detail.
Tables: Reformatted per PLOS ONE (concise captions, footnotes for abbreviations and statistics).
Data availability: dataset prepared; figure/graph underlying values included as Supporting Information.
Funding statement & Role of funder: Updated per journal text.
Permits/Ethics: Methods now include authority name and approval ID; background CH_4 site description clarified.

ORCID: Corresponding author ORCID validated in Editorial Manager.

Below we provide point-by-point responses to Reviewer 1's comments.

Response to Reviewer No.1: We thank the reviewer for the careful assessment and constructive suggestions. We have substantially revised the manuscript to improve clarity, concision, and methodological transparency. Below, we respond to each point and indicate where changes were made.

Comment 1: Data are not sufficient; methods description and citations are too lengthy.
Response: We streamlined the Methods (laser detector description and signal processing) and moved non-essential background into concise citations, while adding missing operational details (e.g., environmental criteria, sampling duration, and background CH_4 handling). We also clarified the experimental design, intake estimation, use of THI, and standardized the statistical reporting across tables/figures. See the revised Methods subsections "Experimental animal, Design and Management," "Milk yield and dry matter intake," "Methane Emission Measurements," and "Statistical analysis." Key insertions are documented below with line-linked citations.

Comment 2: L71–72: What is a crossover design? Provide a detailed description.
Response: We defined the crossover design and described sequences, periods, and alternation to control carry-over: "The study employed a two-sequence AB/BA crossover with four 19-day periods (14-day adaptation, 5-day measurements). During Period 1, groups received SPS vs. TP; from Periods 2–4 treatments were alternated, allowing each animal to serve as its own control."

Comment 3: L74–75: "The comparison between what? Introduce abbreviations at first use."
Response: We now define SPS (silvopastoral system) and TP (traditional pasture) at first mention in Methods (and ensured consistency across the manuscript).

Comment 4: L113–114: "Not necessary; similar issues elsewhere.
Response: Redundant sentences were removed or condensed in Methods; essential operational details were retained (e.g., distance, duration, wind limits). See the tightened LMD description and measurement windows.

Comment 5: L118–123: After listing LMD drawbacks, how did you ensure accuracy?
Response: We added our standardized field protocol: fixed 2 m detector–muzzle distance, ≥240 s per session, wind speed ≤ 2 m s^{-1} with documented direction, single trained operator, and background CH_4 subtraction from upwind readings before/after each session.

Comment 6: Introduction did not summarize the field's research process nor state objectives.
Response: We revised the Introduction to (i) position LMD use under grazing, (ii) emphasize knowledge gaps in humid tropics, and (iii) state explicit objectives (compare SPS vs. TP using standardized LMD protocols; evaluate intensity metrics and behavioral patterns).

Comment 7: L149: "Why alternate treatments? How ensure comparable intake opportunity?
Response: We justify alternation to control carry-over and report forage allowance targeted at 10–12% BW (kg DM/cow/day) to ensure comparable intake opportunities.

Comment 8: L155–170 & Table 2: "Table suggests two plant species in SPS vs. one in TP. What about the trees?"

Response: We added a table footnote clarifying that trees (C. fairchildiana, G. ulmifolia, E. poeppigiana) were present in SPS paddocks but contributed negligibly to diet during the study and were excluded from intake estimates due to early growth stage/canopy.

Comment 9: Figure 1 is hard to understand.
Response: Figure 1 was eliminated, the design used and the treatment scheme in materials and methods are described in detail.

Comment 10: L190–203: Too many methods; how was individual intake calculated?
Response: We clarified that individual DMI combined a double-marker approach (external Cr_2O_3 with recovery rate reported; internal ADL) to estimate total voluntary intake, and an agronomic method (double sampling pre/post-grazing) to partition grass:shrub contributions. References and implementation details are provided.

Comment 11: L215: Relation between measurement time and eating time?
Response: We specified measurement windows aligned with daily routines and diurnal activity (06:00–10:30; 16:30–19:00) and noted concurrent behavioral recording to pair emissions with activity state.

Comment 12: L219: How are these parameters combined with the results?
Response: Behavioral observations were time-stamped and paired with the CH_4 series by session to describe activity-related emission patterns (figure/table summaries). Primary inferential models focus on treatment contrasts (SPS vs TP); behavior is presented descriptively with statistical group comparisons indicated in the figure caption. (See Figure 2 and caption update below.)

Comment 13: L222: Where was background CH_4 measured and how excluded?
Response: Background CH_4 was measured 2 m upwind of the grazing area before and after each session and subtracted from animal-level signals.

Comment 14: Statistical expression in Tables 3 and 5 is not standardized.
Response: We standardized table reporting: means ± SD for descriptive summaries; LSMeans ± SEM for treatment comparisons; n per period/group, and p-values from GLMM. Notes specify tests (e.g., Tukey) and what letters denote.

Comment 15: THI not introduced in Methods. What does it explain?
Response: We added THI methods (calculation and use as a covariate to adjust for environmental variability) and report period-wise THI in Results. (Methods THI paragraph and Table 3 updated; see LMD section context.)

Comment 16: Table 4 should include sample numbers and statistical analysis. Are values averages of two times per day?
Response: Table 4 now states n = 10 cows (5/group) per period, model source (GLMM), and clarifies that reported values are derived from the two daily sessions according to the schedule; p-values are provided.

Comment 17: SEM in Table 5 is not correct.
Response: We verified and corrected SEM; the table now reports LSMeans ± SEM consistent with the GLMM and the caption explains letters/tests. (Revised Table 5.)

Comment 18: Figure 2 lacks statistics; can't determine significance between SPS and TP.

Response: We added letters over bars and a footnote: "Bars with different letters differ significantly (Tukey test, $p < 0.05$)." Error bars are SE. (Revised Figure 2 caption.)

Below we provide point-by-point responses to Reviewer 2's comments.

We sincerely thank Reviewer #2 for the thoughtful and positive assessment. We appreciate your recognition that the topic is both timely and relevant, particularly given the growing global urgency around climate change. Your comment about the scarcity of field-based evidence on livestock GHG emissions in developing-country contexts aligns with our motivation for this work. In revising the manuscript, we have emphasized this contribution more clearly in the Introduction (study relevance and novelty in the Amazonian foothills) and Conclusions (implications for mitigation strategies and inventory refinement in tropical systems). We are grateful for your encouragement and for highlighting the potential of our findings to help fill this critical evidence gap.

Below we provide point-by-point responses to Reviewer 3's comments.

We sincerely thank Reviewer #3 for the careful, line-by-line evaluation and the numerous constructive comments provided in the annotated PDF. Your feedback helped us resolve conceptual ambiguities, strengthen the Conclusions, and align in-text citations and the reference list with PLOS ONE style. We have also undertaken comprehensive language editing to improve clarity, coherence, and consistency across sections. Below we provide point-by-point responses derived from the annotated PDF, indicating where each change was made (section/line/table/figure) and, when useful, quoting the revised text.

Response to Reviewer #3

Comment 1: Methanogenesis in the rumen is a microbial process… Suggests clarifying that methanogenesis is mediated by methanogenic archaea, with minor contributions from methylated substrates.

Response: We agree with this suggestion. The sentence has been revised to highlight the role of methanogenic archaea, specify alternative hydrogen pathways, and clarify CH_4 release via eructation.
Change in manuscript: Methanogenesis in the rumen is a microbial process primarily mediated by methanogenic archaea, which convert hydrogen and CO_2 into CH_4, with minor contributions from methylated substrates. The resulting methane is predominantly released into the atmosphere via eructation (Hai et al., 2025).

Comment 2: If possible rearrange the paragraph on CH_4 quantification methods.
Response: We have reorganized the paragraph for improved readability and logical flow.
Change in manuscript: Respiration chambers, SF_6 tracer technique, and GreenFeed systems are now described in order of accuracy, field applicability, and limitations.

Comment 3: Clarify the GWP of SF_6 (23,500 vs. 25,200) and contextualize its environmental impact.
Response: We clarified the 100-year GWP of SF_6 as reported in the IPCC AR6 (2021), noting inclusion of climate-carbon feedbacks, and acknowledged the small mass used in tracer studies.
Change in manuscript: …SF_6 is a potent greenhouse gas, with a 100-year global warming potential of 25,200 (including climate–carbon feedbacks; IPCC, 2021). Although the per-molecule impact is high, the actual mass released in tracer studies is minimal (micrograms per animal per day), but the precautionary principle still warrants consideration.

Comment 4: Secondary metabolites should be explained (how they reduce CH_4).
Response: We revised the introduction to briefly describe their mechanisms (inhibition of methanogens and alternative hydrogen sinks).
Change in manuscript: …secondary metabolites such as tannins and saponins, which may reduce CH_4 emissions by inhibiting methanogenic archaea or redirecting hydrogen toward alternative fermentation pathways (Patra & Saxena, 2010; Jayanegara et al., 2012).

Comment 5: Avoid repetition of 'the present study' and 'findings'.
Response: We modified the sentence to use active voice and concise phrasing.
Change in manuscript: This study evaluated the use of the laser methane detector (LMD) to estimate enteric CH_4 emissions in dairy cows grazing a silvopastoral system and a grass monoculture in the Colombian Amazonian foothills.

Comment 6: Provide vegetation coverage and forage species in study area description.
Response: We added vegetation and forage information to the site description.
Change in manuscript: The study area is located in an equatorial tropical rainforest with a Köppen climate classification of Af [26]. It is characterized by a mosaic of natural pastures and secondary forest patches, where the dominant livestock forages are *Urochloa decumbens* and *Urochloa humidicola*.

Comment 7: Please state standards for 19-day periods (14 adaptation, 5 evaluation) and design type.
Response: We added references and specified the crossover design.
Change in manuscript: The study employed a crossover design (Kung-Jong, 2016), with four 19-day periods (14 days adaptation, 5 days evaluation; Machado et al., 2016).

Comment 8: Table 1 (animal characteristics) seems not applicable; supplementary data?
Response: We moved this table to Supplementary Material (S1 Table), cited in Methods.
Change in manuscript: Added reference in Animals and Experimental Design: Animals were allocated into two homogeneous groups… (see S1 Table).

Comment 9: Table 2 (nutritional composition): Is this supplementary?
Response: We kept this table in the main text, as it is essential for interpreting CH_4 results. It describes forage composition in both systems and provides the nutritional context of the experiment.
Change in manuscript: Retained as Table 2 with clarification in Materials and Methods.

Comment 10: THI not mentioned in Methods.
Response: We separated and clarified the THI subsection in Methods.
Change in manuscript: Temperature-Humidity Index (THI) was calculated using NRC (1971). Values were later analyzed in relation to CH_4 emissions.

Comment 11: Add more points from Table 4 in Results (THI).
Response: We expanded the description of THI values without interpretation.
Change in manuscript: …ranging from 75.95 in the first period to 87.19 in the fourth period (Table 4). Mean values remained above 75 throughout, with the highest values in the last two periods.

Comment 12: Behavioral results not supported by Methods.
Response: We created a new subsection Animal Behavior Recording in Methods.
Change in manuscript: Based on Patiño Pardo (2003), with categories PST, RM, DE, OC, OR.

Comment 13: Table 4 title too long.
Response: Shortened for clarity.
Change in manuscript: Table 4. Temperature-Humidity Index (THI) and daily methane emissions in cows grazing silvopastoral (SPS)

Comment 14: Conclusions: Review. Do not mention secondary metabolites if not discussed.
Response: We revised conclusions, removed secondary metabolites, softened wording, and focused on results.
Change in manuscript: …reduced enteric methane emissions per unit of milk yield compared with a grass monoculture… The LMD proved to be a practical tool… These results highlight the potential of SPS for mitigating GHG in tropical livestock.

Below we provide point-by-point responses to Reviewer 4's comments.

We are grateful to Reviewer #4 for the positive assessment and the clear, actionable guidance—especially regarding the lack of inter-study standardization for LMD measurements. Your comments prompted us to sharpen the framing of our findings and to correct several presentation details. Below we provide point-by-point responses to your comments, indicating where each change was made and the nature of the revision.

We appreciate your careful review; these refinements have improved the manuscript's methodological rigor, statistical transparency, and interpretive caution regarding LMD-derived estimates.

Comment 1: Emphasis on standardization and scope of inference. We now explicitly state that, given the absence of fully standardized LMD acquisition/processing protocols, our inferences focus on within-study contrasts (SPS vs. TP) under a harmonized field protocol rather than on direct cross-study comparisons of absolute values. Revisions appear in the Abstract (last sentence) and Discussion (opening paragraph).

Comment 2: Table label correction. "SSP" has been corrected to "SPS" in Table 5 (now Table numbering aligned after edits).

Comment 3: Significance in text and Figure 2. We added p-values/letter groupings to indicate statistical significance in the Results text (around former line 316) and in the Figure 2 caption ("Bars with different letters differ significantly; Tukey test, $p < 0.05$"), and ensured error bars and sample size are reported consistently.

Comment 4: Citations for *P. discolor* nutritive content. We added/verified citations supporting crude protein, fiber fractions, and energy estimates for P. discolor in Methods (Diet/Composition) and Discussion where nutrient values are discussed.

Comment 5: Wording refinement ("enhance" to "alter"). As suggested, we changed "enhance" to "alter" where describing potential effects on fermentation pathways, to maintain a mechanistically cautious tone (Discussion, effects of diet on fermentation).

We thank the Editor and Reviewers again. We believe the revisions substantially improve clarity, methodological rigor, and relevance for humid tropical grazing systems and for the ongoing

discussion on field-based LMD measurements. We remain at your disposal for any further adjustments.
The funders had no role in study design, data collection and analysis, decision to publish, or preparation of the manuscript.

On behalf of all authors,

Juan Pablo Narváez-Herrera
Joaquín Angulo-Arizala, Wilson Andrés Barragán-Hernández, and Liliana Mahecha-Ledesma
Universidad de Antioquia

Corresponding author: Liliana Mahecha-Ledesma
liliana.mahecha@udea.edu.co
