# Supplementary material for: Estimation of enteric methane emissions in dairy cows under grazing a silvopastoral system and a grass monoculture in the Colombian Amazonian foothills
Source: PLoS One. 2026 Jan 30;21(1):e0337719. doi: 10.1371/journal.pone.0337719 (PMC12857995; doi:10.1371/journal.pone.0337719)
Supplement: Attachment — Submitted filename: Review Report.docx [file pone.0337719.s003.docx]

Dear Editors

Thank you very much for your invitation as a potential reviewer to review the manuscript titled “Estimation of Enteric Methane Emissions in Dual-purpose Cows under grazing a Silvopastoral system and a Grass Monoculture in the Colombian Amazonian Foothills”

**Authors:** Liliana Mahecha-Ledesma, Juan Pablo Narvaez Herrera, Joaquín Angulo-Arizala, Wilson Andrés Barragán-Hernández, Liliana Mahecha-Ledesma

**Journal name**: PLOS ONE

**Manuscript number**: PONE-D-25-33619

**Date Received**: Aug 14 2025

**General comment forwarded:** The topic is timely and relevant, addressing important aspects of livestock production system and productivity improvement strategies in changing global climate effect. Nowadays clime change issue is becoming the global concern. There is limited scientific evidence which indicates the current status of livestock GHG emissions particularly in developing countries; the findings may fill this gap in indicating effective mitigation strategies. However, I have suggestions to improve the manuscript before it is suitable for publication:

1. Abstract: Please clearly stat the research area and articulating the study's novelty and relevance to the scientific community.

- Why you focus on laser methane detector (LMD) than other method?
- An experimental animal was classified based on feed type they consume or based on animal activity? Why?
- Limitation of the study or strong side

2. Introduction: The background section provides useful global and study area context but lacks full information and cohesion.

- Some paragraph lack citation, old citation
- Other study done so far related with laser methane detector (LMD) method was not indicated
- Advantage and disadvantage of each production system based on feed resource availability, management system….

3. Materials and methods: This part addressed well but need further clarify on the rationale behind the experimental design and sampling periods.

- Please use recent literature, olds citation (1966)

4. Results and Discussions: it is better to indicate future research under conclusions and recommendations part
5. Conclusions: This section is well addressed but it needs some revision

- Please relate your study finding with the objectives of the study.
- Discuss its broader implications for policy makers
- Include study limitations
- How do you relate your finding or suggestion with pastoral production system

6. Grammatical error, consistency or coherence and proof reading: it hinders the overall quality of the paper.

- Proper citation and referencing: Please careful check the authors cited in the body of the manuscript is properly referenced under references part of the manuscript.
- Lack order 108, 113

Finally, the manuscript has strong potential and addressing these points will improve its clarity, and provide relevant information about the enteric methane gas emissions of livestock managed under different grazing system and its mitigation strategy.

I look forward to seeing a revised version.
